# Conflicting Roles of 20-HETE in Hypertension and Stroke

**DOI:** 10.3390/ijms20184500

**Published:** 2019-09-11

**Authors:** Shashank Shekhar, Kevin Varghese, Man Li, Letao Fan, George W. Booz, Richard J. Roman, Fan Fan

**Affiliations:** 1 Department of Neurology, University of Mississippi Medical Center, Jackson, MS 39216, USA; 2 Department of Pharmacology and Toxicology, University of Mississippi Medical Center, Jackson, MS 39216, USA

**Keywords:** cytochrome P450, hypertension, stroke, cerebral autoregulation, endothelial dysfunction, vascular remodeling, vascular inflammation, blood-brain barrier, angiogenesis

## Abstract

Hypertension is the most common modifiable risk factor for stroke, and understanding the underlying mechanisms of hypertension and hypertension-related stroke is crucial. 20-hydroxy-5, 8, 11, 14-eicosatetraenoic acid (20-HETE), which plays an important role in vasoconstriction, autoregulation, endothelial dysfunction, angiogenesis, inflammation, and blood-brain barrier integrity, has been linked to hypertension and stroke. 20-HETE can promote hypertension by potentiating the vascular response to vasoconstrictors; it also can reduce blood pressure by inhibition of sodium transport in the kidney. The production of 20-HETE is elevated after the onset of both ischemic and hemorrhagic strokes; on the other hand, subjects with genetic variants in *CYP4F2* and *CYP4A11* that reduce 20-HETE production are more susceptible to stroke. This review summarizes recent genetic variants in *CYP4F2,* and *CYP4A11* influencing 20-HETE production and discusses the role of 20-HETE in hypertension and the susceptibility to the onset, progression, and prognosis of ischemic and hemorrhagic strokes.

## 1. Introduction

Hypertension is a prevalent chronic disease that affects approximately 75 million people in the U.S. and an estimated cost of $48.6 billion annually [1]. Unlike hypertension, which takes years to manifest clinically; stroke presents with sudden and devastating neurological consequences. Stroke is one of the major risk factors of mortality and causes more survivors with long-term disability than other risk factors [2]. Hypertension is the most common modifiable risk factor of stroke. It acts through various mechanisms affecting vascular smooth muscle cells (VSMCs), endothelial dysfunction, oxidative stress, changes in cerebral blood flow, and inflammation [3,4].

Numerous animal and clinical studies have indicated the importance of genetic and environmental factors to the pathogenesis of stroke. A genome-wide association study (GWAS) demonstrated that genetic variation in the Notch3 receptor is linked to cerebral autosomal dominant arteriopathy with subcortical infarcts and leukoencephalopathy (CADASIL), or cerebral autosomal-recessive arteriopathy with subcortical infarcts and leukoencephalopathy (CARASIL) [5]. Single nucleotide polymorphisms (SNPs) or mutations have been linked to increased risk of ischemic (i.e., *COL3A1, GLA*) or hemorrhagic stroke (i.e., *APP, COL4A1*) [6]. Many of these same SNPs also are associated with hypertension. For example, a recent clinical study with 18,000 ischemic stroke patients and 70,000 controls identified SNPs in *SH2B3* and *ALDH2* linked to ischemic stroke, which were also associated with blood pressure [6]. Variants in *CYP4F2* and *CYP4A11* have been repeatedly linked to hypertension, stroke, and cardiovascular disease [7,8,9,10,11].

**Figure 1:**
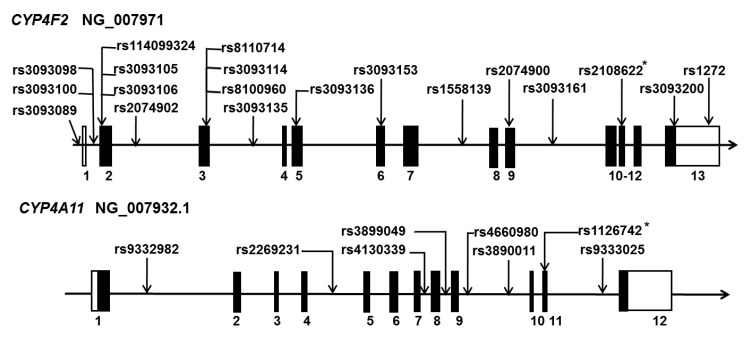
Genetic polymorphisms in human *CYP4F2* and *CYP4A11***.** Localization of single nucleotide polymorphisms (SNPs) in genomic DNA of *CYP4F2* (upper panel, NG_007971) and *CYP4A11* (lower panel, NG_007932.1). Numbers indicate the order of exons. Solid portions of exons indicate transcription regions. Open frames on exons indicate non-transcription regions of *CYP4F2* and *CYP4A11*. Published RefSNPs (rs) numbers linked with hypertension and stroke are listed. Asterisks (*) indicate that the SNPs are also associated with other diseases, including cardiovascular disease and kidney transplantation.

CYP4F2 and CYP4A11 are the two most potent enzymes to catalyze the metabolism of arachidonic acid (AA) to 20-hydroxy-5, 8, 11, 14-eicosatetraenoic acid (20-HETE) in humans [12]. A number of SNPs in *CYP4F2* and *CYP4A11* have been reported to play an essential role in the development of hypertension and stroke in several populations, as summarized in Figure 1. These include rs2108622 (V433M) and rs1558139 in *CYP4F2* in Japanese, Australian, Swedish, Chinese, South Indian, and American subjects [11,13,14,15,16,17,18,19] and rs1126742 (F434S), rs9333025, and rs389011 in *CYP4A11* in Chinese, American, Japanese, and African subjects [11,20,21,22,23,24].

Among these SNPs, rs2108622 (V433M, *CYP4F2*) was confirmed to reduce 20-HETE production in vitro [25,26], and increase urinary 20-HETE excretion [14], and rs1126742 (F434S, *CYP4A11*) was first reported to reduce 20-HETE synthase activity [27]. Saito et al, reported that five (rs12564525, rs2056900, rs2056899, rs4926581, and rs2405599) of 10 variants in *CYP4A11* or *CYP4A22* identified in the Pharmacogene Variation Consortium (https://www.pharmvar.org/) markedly reduced activity when expressed in COS-7 cells [28]. However, the functional significance of these observations has not been identified. In animal studies, Dahl salt-sensitive (SS) rats rapidly develop severe hypertension when fed a high salt diet. This strain has a deficiency in the expression of CYP4A enzymes and the formation of 20-HETE. They displayed cerebral vascular dysfunction and enhanced blood–brain barrier (BBB) leakage, which may increase the susceptibility to the development of stroke [29,30]. The available evidence seems to suggest that reduced 20-HETE levels may predispose to the development of stroke, although alterations in 20-HETE production associated with many of the human *CYP4F2* and *CYP4A11* SNPs have not been defined. On the other hand, numerous studies in human and animal models indicate that 20-HETE production is enhanced following traumatic brain injury (TBI) and ischemic and hemorrhagic stroke [31,32,33]. Elevations in 20-HETE contribute to an acute fall in cerebral blood flow (CBF) and delayed vasospasm following subarachnoid hemorrhage (SAH). Inhibitors of the formation of 20-HETE reduce infarct size following ischemic stroke and reverse vasospasm following SAH [7,33]. Similarly, inhibition of 20-HETE prevents BBB leakage, reduces cerebral edema, and is beneficial to the outcomes following TBI [31,32]. Ischemic stroke patients who have both rs9333025 GG (*CYP4A11*) and rs2108622 GG (*CYP4F2*) variants have higher plasma 20-HETE levels [34]. Spontaneously hypertensive rats (SHR) that have elevated endogenous 20-HETE production are more susceptible to ischemic stroke. Elevated 20-HETE levels are also associated with worse prognosis in SAH patients [17,33]. This evidence suggests that elevated 20-HETE participates in the severity of ischemic and hemorrhagic strokes.

Indeed, the link between 20-HETE and hypertension is complicated as 20-HETE promotes the effects of vasoconstrictors to increase blood pressure in some models but reduces blood pressure by inhibition of sodium transport in the kidney in other models [12,33]. The complicated relationship between 20-HETE and stroke is thus expected, not only because of the conflicting roles of 20-HETE, but also because of the complexity of the onset, development, and prognosis in different types of stroke. This review addresses the conflicting roles of 20-HETE in hypertension and stroke by dissecting the molecular mechanisms of 20-HETE and its contribution at various stages of ischemic and hemorrhagic stroke.

## 2. 20-HETE in Hypertension

### 2.1. 20-HETE

20-HETE is an AA metabolite catalyzed by enzymes of the CYP4A and CYP4F families. In humans, CYP4A11, CYP4A22, CYP4F2, and CYP4F3 are responsible for generating 20-HETE through ω-hydroxylation. CYP4F2 and CYP4A11 are the two most potent isoforms. [35]. The major isoforms of these enzymes in rats, CYP4A1, CYP4A2, CYP4A3, CYP4A8, CYP4F1, and CYP4F4 are found in the liver, kidney, brain, blood vessels, and other tissues. Among these, CYPA1 is the most active isoform in the production of 20-HETE [7,33]. In mice, there are several isoforms of CYP4A expressed, including CYP4A10, CYP4A12A, CYP4A12B, and CYP4A14, but only CYP12A can produce 20-HETE [12]. 20-HETE can be inactivated by cyclooxygenase-2 (COX2) after conversion to 20-hydroxy-PGE. It can also be metabolized and inactivated by alcohol dehydrogenase (ADH) to 20-carboxy-eicosatetraenoic acid (20-COOH-HETE) which can be further converted to the 18-dicarboxylic acid and 16-dicarboxylic acid through beta-oxidation. The actions of 20-HETE can also be terminated by metabolism to a glucuronide by uridine 5’-diphosphoglucuronosyltransferase (UGT) [7].

20-HETE exhibits a wide variety of functions essential to the physiology and pathogenesis of many diseases. It acts as a natriuretic agent in the kidney, serves as an autocrine factor, and potentiates the effects of vasoconstrictive agents. It also promotes renal oxidative stress, endothelial dysfunction, vascular inflammation, and activates the renin-angiotensin-aldosterone system (RAAS), especially in the vascular endothelium [12]. Moreover, 20-HETE plays a role in angiogenesis, cell proliferation, vascular remodeling, and restenosis [33,36]. In addition, by suppressing thromboxane formation, 20-HETE inhibits platelet aggregation [37]. Administration of angiotensin II (ANG II) stimulates phospholipase A2 (PLA2) and releases AA resulting in enhanced 20-HETE production [38]. Induction of heme oxygenase (HO) reduces, and administration of dihydrotestosterone (DHT) elevates, 20-HETE production in the kidney and vasculature [12]. Carbon monoxide (CO) and nitric oxide (NO) act as inhibitors of 20-HETE [8].

Two 20-HETE receptors have been discovered recently. A chemokine CCL5 (RANTES) receptor, G-protein-coupled receptor GPR75 (Gq), was identified to bind to 20-HETE leading to endothelial dysfunction [39]. We reported that GPR75 is highly expressed in the brain and cerebral vasculature at protein levels in rats [40], and it is highly expressed at mRNA levels in the brain in humans (Human Protein Atlas: https://www.proteinatlas.org/ENSG00000119737-GPR75/tissue); however, the exact cellular localization and roles of GPR75 in the brain are yet to be determined [41]. It is interesting that CCL5 is highly expressed in the cerebral vasculature in Alzheimer’s patients [42], which prevents amyloid β-induced neuronal cell death via activation of GPR75 [43]. High doses of 20-HETE also activate GPR40 in the pancreatic islet cells to regulate insulin secretion [44], but epoxyeicosatrienoic acids (EETs) activate GPR40 in endothelial, smooth muscle cells and arteries resulting in vasodilation [45]. Nevertheless, whether GPR75 and GPR40 play a role in the vascular effects of 20-HETE in stroke in unknown.

### 2.2. 20-HETE in Hypertension

20-HETE has both pro- and anti-hypertensive actions. Here, we mainly discuss the conflicting roles of 20-HETE in the kidney and vasculature in the control of blood pressure.

#### 2.2.1. 20-HETE and the Kidney

20-HETE reduces sodium transport by inhibition of Na^+^/K^+^-ATPase activity and internalization of the sodium–hydrogen exchanger 3 (NHE3) in the proximal tubule (PT). It also blocks Na^+^/K^+^-ATPase, and potassium channels and limits sodium and potassium uptake via the Na–K–Cl cotransporter (NKCC2) in the thick ascending loop of Henle (TALH) [8,46]. 20-HETE also interacts with ANG II, dopamine, endothelin, and parathyroid hormone, and contributes to their natriuretic effects in the PT. The production of 20-HETE was elevated when renal perfusion pressure was increased, indicating its role in pressure natriuretic response [8].

#### 2.2.2. 20-HETE and the Vasculature

In the vasculature, 20-HETE constricts VSMC and contributes to the responses to stretch and vasoconstrictors such as ANG II, serotonin (5-HT), and endothelin by blocking calcium-activated potassium (BK) channel activity and enhancing calcium influx through voltage-gated L-type calcium channels. It promotes cell proliferation and endothelial dysfunction and is associated with vascular inflammation [7]. Moreover, a 20-HETE receptor, GPR75, has been linked to endothelial dysfunction [39]. 20-HETE augments constriction of VSMCs by enhancing calcium entry via activation of mitogen-activated protein kinases (MAPKs), protein kinase C (PKC), Rho-associated protein kinase (ROCK), and tyrosine-kinases [33]. 20-HETE enhances the myogenic reactivity and autoregulation of CBF and renal blood flow [7,29]. In the kidney, 20-HETE increases constriction of the afferent arteriole (Af-art) in response to elevations in perfusion pressure [47]. Blockade of 20-HETE formation diminished the vasoconstrictor response to both elevations in transmural pressure and is thought to be the mediator of tubuloglomerular feedback responses (TGF) [47]. Indeed, inhibitors of 20-HETE have been shown to block TGF-mediated constriction of the Af-art of isolated perfused glomeruli with an intact macula densa to elevations in sodium concentration in the tubular perfusate. 20-HETE inhibitors also blocked TGF in vivo [7,47].

20-HETE has been reported to increase the expression of angiotensin-converting enzyme (ACE) in the endothelium. Upregulation of the formation of 20-HETE in the vasculature in response to administration of androgens or *Cyp4a12* transgenic and *Cyp4a14* knockout (KO) mice produces 20-HETE dependent hypertension that is mediated in part through increases in the expression of ACE in the vascular endothelium. Indeed, 20-HETE induced hypertension and vascular hypertrophy can be blocked by administration of ANG II receptor type 1 (AT1) receptor antagonists [7].

Previous studies have indicated that the expression of CYP4A enzymes and the production of 20-HETE in the kidney and vasculature are reduced in SS rats [29,30]. SNPs in *Cyp4a* on chromosome 5 cosegregate with salt-sensitive hypertension in an F2 cross of SS and Lewis rats [48]. Moreover, a deficiency in 20-HETE is thought to reset the pressure-natriuretic relationship and enhance sodium transport in the PT and TALH. Induction of the formation of 20-HETE with fibrates or transfer of wild type (WT) *Cyp4a* genes in congenic strains improves sodium excretion and reduces blood pressure [8,49]. In contrast, the expression of CYP4A and production of 20-HETE are elevated in the vasculature of the SHR. Inhibitors of the formation of 20-HETE markedly reduce blood pressure in this strain [8,49].

## 3. 20-HETE in Stroke 

Each year around 750,000 strokes occur in the USA, with 85% of that comprised of the ischemic type and 15% of the hemorrhagic type. Stroke results in a large number of patients with moderate to severe disability. Hypertension and diabetes are the most common factors that predispose patients to stroke [1,2].

Ischemic stroke occurs when a propagating clot, inherent plaque, or a damaged vessel suddenly occludes the blood supply in the specific region of the brain. Various ischemic stroke rodent models have been established to study different pathophysiologies and the immediate effect of occlusion [50]. Following occlusion of a cerebral vessel, there is a failure of ATP-dependent pump resulting in accumulation of intracellular sodium and calcium ions and depletion of intracellular potassium ions in the ischemic core. These changes cause cellular acidification and cell swelling, damaging of organelles including mitochondria, the release of proteolytic enzymes and inflammatory mediators, and eventually promote neuronal cell death. The degree of cerebral ischemic damage is augmented by the release of other factors such as glycine, glutamate, dopamine, 5-HT, and free fatty acids (FAA). FAA are used as substrates for the formation of neuronal lipid peroxides, which affects the metabolism and oxygen utilization of cells. Ischemia also promotes the release of other fatty acids such as the 20-HETE substrate AA, leukotrienes, and eicosanoids, which produce free radicals and further enhance leukocyte recruitment and cellular damage [7].

Subarachnoid hemorrhage (SAH), the most common type of hemorrhagic stroke, results from bleeding in the arachnoid space and clot formation. The cause of non-traumatic SAH and intracerebral hemorrhage (ICH) is an aneurysmal rupture. Hypertension has been implicated as one of the main risk factors for aneurysmal formation and rupture [51,52]. Acute cerebral ischemia (ACI) occurs within hours after intracranial bleeding occurs. There is also increased intracranial pressure dependent on the volume of bleed that further compromises cerebral perfusion during the ACI period, followed by delayed cerebral ischemia (DCI) due to a second peak if vasospasm, which can extend up to 21 days in some patients. Almost a third of patients who develop DCI suffer from permanent neurological damage [53,54,55].

### 3.1. 20-HETE in the Susceptibility of the Onset of Stroke

Emerging evidence demonstrates that several SNPs in *CYP4F2* and *CYP4A11* are associated with hypertension and stroke in several populations. A large urban-based population study in Sweden suggested there may be an increased risk of stroke, especially in males in the presence of the *CYP4F2* rs2108622 (V433M) variant [15]. In Japanese men, the presence of the *CYP4F2* rs2108622 G allele was associated with an increased risk of ischemic stroke [13]. Genetic polymorphisms in *CYP4F2* were also found in Chinese [18,19], South India [16], Australian [14], and American [17] patients and were associated with an increased incidence of hypertension and both ischemic and hemorrhagic strokes. The SNPs of rs1126742 (F434S), rs9333025, and rs389011 in *CYP4A11* were found to be associated with hypertension and stroke in American [24,27], Chinese [20,56], and Japanese [21,22] populations. These reports suggest that 20-HETE plays an important role in increasing susceptibility to the onset of stroke, especially in hypertensive patients. However, it remains to be determined whether this increased susceptibility is induced by lower or higher 20-HETE levels.

Two clinically relevant SNPs have been confirmed to reduce the formation of 20-HETE: *CYP4F2* rs2108622 (V433M) and *CYP4A11* rs1126742 (F434S) [25,26,27]. The observation that these SNPs are associated with an increased incidence of stroke could be explained by the fact that reduced 20-HETE diminishes the vasoconstrictive capability of cerebral vessels, which causes impaired myogenic response and autoregulation of cerebral blood flow. Such cerebral vascular dysregulation could transmit higher perfusion pressure to downstream capillaries, especially in association with hypertension. Enhanced pressure could distend the capillaries and damage BBB integrity resulting in increased permeability and bleeding. Further, with time, elevated perfusion pressure might increase capillary rarefaction and focal ischemia. This hypothesis is consistent with the results of our recent study. We found that SS rats with a 20-HETE deficiency exhibited impaired cerebral vascular autoregulation and BBB leakage, which was rescued in a CYP4A1 transgenic rat on the SS genetic background that restored vascular production of 20-HETE [29].

In contrast, ischemic stroke patients with both *CYP4A11* (rs9333025) and *CYP4F2* (rs2108622) SNPs exhibit elevated plasma 20-HETE levels [56]. Subjects of 322 minor ischemic stroke in a multicenter prospective observational study in China had levels of 20-HETE as high as 1,675 pmol/L [57]. These findings suggest that elevated 20-HETE production may promote the development of stroke, which at first glance seems contradictory to a role of reduced 20-HETE levels in stroke. However, animal studies in the SHR rat model are consistent with these reports since this hypertensive rat model exhibits higher 20-HETE levels and enhanced infarct size following transient ischemia and reperfusion injury. Moreover, since 20-HETE promotes oxidative stress, endothelial dysfunction and vascular inflammation, the hypothesis that elevated 20-HETE increases the susceptibility to the onset of stroke also seems logical. Although GPR75 has been identified as a 20-HETE receptor and contributes to endothelial dysfunction, it remains to be determined if the receptor plays a role in 20-HETE-related cerebrovascular function or in stroke [39,41].

Indeed, there is not enough evidence to determine whether 20-HETE is beneficial or detrimental to the onset of stroke. In fact, the controversial results between the available human and animal studies cannot be solely explained by the function of 20-HETE. The pathogenesis of hypertension and stroke are multi-factorial, including genetic and environmental. It is also possible that alterations in 20-HETE production caused by SNPs in *CYP4A11* and *CYP4F2* interplays with other molecules in the AA-CYP4A/4F-20-HETE pathway and changes the production of other AA metabolites catalyzed by cyclooxygenase (COX) and lipoxygenase (LOX).

### 3.2. 20-HETE in the Progression of Stroke

#### 3.2.1. 20-HETE in the Progression of Ischemic Stroke

The production of 20-HETE is elevated in the plasma of ischemic stroke patients [58]. This observation is consistent with results obtained from animal studies using the middle cerebral artery occlusion (MCAo) model in rodents [7,33]. The levels of 20-HETE in MCAo rats are not only elevated in plasma but also cerebral tissues. As discussed above, the enhanced 20-HETE production after ischemic stimulation could be multifactorial, including accumulation of intracellular sodium and membrane depolarization to trigger calcium influx, and the release of AA. Elevated levels of 5-HT and glutamate also stimulate phospholipase and the release of AA following reperfusion. This view was confirmed in animal studies that revealed that inhibition of 20-HETE significantly reduced infarct volume and improved the neurological outcomes by reducing oxidative stress and apoptosis in neurons, rather than through a reduction of blood flow in the penumbral regions by 20-HETE [7,33,59,60,61]. In addition to increasing infarct size, elevated 20-HETE levels in mild ischemic stroke patients were shown to be associated with neurological dysfunction (ND). In this study, 72% of patients were found to have ND within the first 24h of admission [57]. Chronic administration of 20-HETE inhibitors in MCAo rats reduced neurological deficits [44]. 20-HETE synthase was elevated on the ipsilateral side 7 days after brain ischemic injury in a positron emission tomography (PET)-based study [62] suggesting upregulation of CYP4A and CYP4F isoforms might be involved in angiogenesis and neuroinflammation after stroke. Moreover, in a study analyzing carotid plaque characteristics, 20-HETE was found to be significantly higher in a patient with atheroma plaques compared to other metabolites [63]. Whether the 20-HETE content in the atheromatous plaque has any consequence on stroke outcome is still unclear, however, genetic variations in CYP450 isoforms have also been associated with plaque stability in patients with ischemic stroke [64].

#### 3.2.2. 20-HETE in the Progression of Hemorrhagic Stroke

After the aneurysmal rupture, blood spreads into the arachnoid space and the perivascular spaces. The direct effect of blood and its components results in the sentinel headache and the initial cascade of pathological effects which causes the early neurological changes [65]. The severe headache may proceed with acute vasospasm contributing to ND on the first two days [54]. In most cases, it will be followed by delayed vasospasm, which takes days to weeks and has the most devastating consequences for SAH patients [7,17].

The acute injury in SAH results from the direct mechanical damage of bleeding [65]. The initial drop in the cerebral blood flow is also due to acute vasospasm and is correlated with the amount of the hemoglobin (Hb) in the cerebrospinal fluid (CSF) that was validated by the injection of clotted blood or oxyhemoglobin into the CSF [66]. The acute vasospasm is induced by the release of vasoconstrictors such as 5-HT and ATP from the clots, scavenging of the potent vasodilator NO by Hb, as well as the release of AA stimulated by 5-HT-activated PLA2 that produces 20-HETE [7,67]. The decrease in NO levels also stimulates CYP4A expression and in turn, 20-HETE production. Blocking the synthesis of 20-HETE with HET0016 prevents the fall in rCBF, and reduces the vasoconstrictor response of isolated MCA by >60% after SAH [68]. The elevated 20-HETE levels contribute to delayed vasospasm. DCI occurs in 30 to 40% of SAH patients [54]. The pathophysiologic processes responsible for DCI include impaired cerebral autoregulation, cortical spreading ischemia (CSI), microvascular thrombosis, and BBB disruption [33,54]. In animal SAH models, including rats and dogs, DCI was associated with increased levels of 20-HETE in CSF, and 20-HETE inhibitors reversed delayed vasospasm in these models [69,70].

Similarly, elevated levels of 20-HETE in CSF after SAH in human has been reported [17]. SAH is associated with activated PKC, MAPK, and ROCK, as well as diminished potassium channel activity, all of which results in depolarization and activation of VSMC. Both acute and delayed vasospasm following ischemic stroke could be attenuated with blockage of the actions of vasoconstrictors, including 20-HETE, 5-HT receptor, thromboxane, and ET_A_ receptor, as well as inhibition of PKC, MAPK, and ROCK [7]. 

CSI, induced by the wave of neuronal depolarization or cortical spreading depolarization (CSD), is thought to play a role to brain damage in SAH patients by resulting in impaired neurovascular coupling and reduced cerebral blood flow in SAH [71]. The increased CSD has been associated with elevated 20-HETE synthesis and reduced cerebral blood flow in rat models, and 20-HETE inhibitors resulted in a neuroprotective effect and reduction in edema [71,72].

Very little is known about the relationship between 20-HETE and ICH. Administration of 20-HETE inhibitor reduced the hemorrhagic volume, the neurological deficit, and edema in ICH via reducing inflammatory response and ROS production without angiogenesis [73,74].

**Figure 2:**
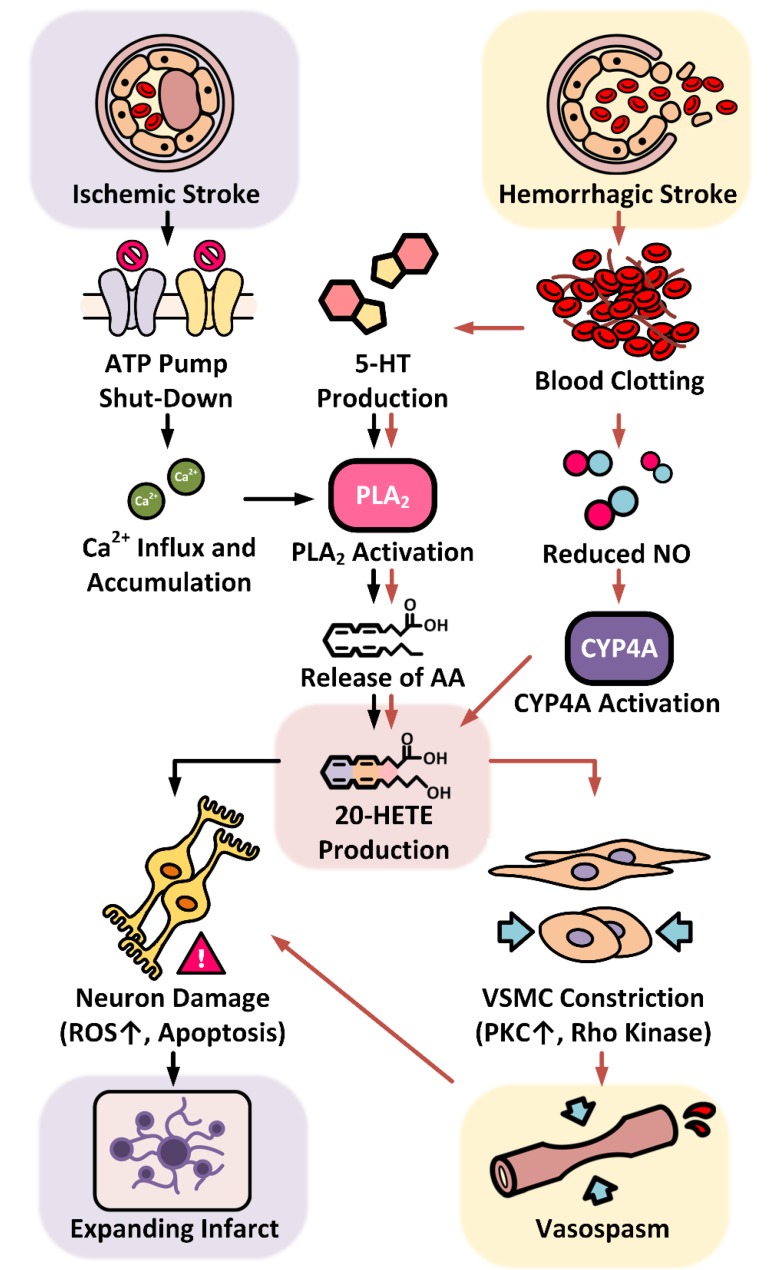
Role of 20-HETE in the progression of ischemic and hemorrhagic strokes. Ischemic stroke leads to hypoxia and ATP depletion, and failure of the ATPase ion pumps within the ischemic core. These effects result in accumulation of intracellular sodium and calcium ions. Enhanced calcium influx activates phospholipase A2 (PLA2) to release arachidonic acid (AA) from the membrane, which is converted into 20-hydroxyeicosatetraenoic acid (20-HETE) by CYP4A enzymes. Clotting blood formed after hemorrhagic stroke releases free hemoglobin and serotonin (5-HT). Free hemoglobin scavenges nitric oxide (NO), and the fall in NO level promotes the activity of CYP4A enzymes. 5-HT stimulates PLA2 and releases AA to increase 20-HETE production. The rise of 20-HETE acts on vascular smooth muscle cells leading to vasospasm, further restricting the blood flow to the stroke site and inducing neuron damage. 20-HETE also affects neurons directly by increasing oxidative stress causing expansion of the infarct after ischemic stroke.

As summarized in Figure 2, it has been well established that elevated 20-HETE levels play a detrimental role in the progression of ischemic and hemorrhagic strokes and worsens neurological outcomes in human and animal SAH models.

### 3.3. 20-HETE in the Prognosis of Stroke

Approximately 25% of stroke patients die from this disease. Ten percent of ischemic stroke patients completely recover, but the outcomes after hemorrhagic stroke are generally worse than for ischemic stroke [1,2]. Long-term complications of stroke include disability and dementia. Prognosis of stroke not only depends on the type of stroke and the degree and duration of stroke but is also influenced by genetic and environmental factors.

BBB breakdown worsens the ischemic injury and promotes inflammation after stroke. Endothelial cells, pericytes, and astrocytes all play essential roles in maintaining the integrity of BBB. These cells all express CYP4A and produce 20-HETE [7,40,75]. A study using an MCAo obese ob/ob mouse demonstrated that increased BBB leakage enhanced ischemic brain injury by inducing astrocyte end-foot swelling and endothelial vesicles in association with vessel lumen collapse [76]. Low 20-HETE formation in SS rats has been shown to increase BBB leakage due to impaired myogenic response and CBF autoregulation, resulting in increased capillary pressure [29]. Impaired myogenic response and vascular leakage in hypertensive patients has been suggested to accelerate white matter damage and increase the risk of lacunar infarctions and later cognitive decline [77]. We also found that there is an association of SNPs in *CYP4F2* and *CYP4A11* with mild cognitive impairment and dementia-related endophenotypes including brain volumes and performance on cognitive tests, although only two of those SNPs were reported to reduce 20-HETE production [11]. On the other hand, inhibition of 20-HETE formation reduced BBB permeability in a rat TBI model [31]. This could be due to endothelial dysfunction, but whether and how 20-HETE acts on pericytes and astrocytes to affect BBB integrity has not been studied.

Long-term recovery from stroke also depends on the degree of angiogenesis [78]. The role of 20-HETE in angiogenesis is now well established. Sa et al. first reported that fibroblast growth factor 2 (FGF2) activates PLA2 via the p42 MAPK pathway which leads to a release of AA from membrane phospholipids which further stimulates CYP4A to produce 20-HETE [79]. Chen et al. reported that hypoxia and vascular endothelial growth factor (VEGF) induced *CYP4A11* gene expression and increased 20-HETE production in endothelial progenitor cells (EPC) in vivo. In contrast, the angiogenesis was reduced by local inhibition of the 20-HETE system, suggesting 20-HETE has a vital role in EPC medicated angiogenesis [80]. 20-HETE inhibitors have been reported to block the neovascularization of the cornea and the implanted glioblastoma cell (solid tumors) [81]. Various other studies using 20-HETE inhibitors have observed similar reduced vascularization and growth of rat 9L and human U251 glioma cells implanted in the brain of nude rats [7]. The reason is likely due to upregulation of CYP4A as reported by Guo et al., which enhanced phenotypic proliferation in both in vivo and in vitro experiments [82,83]. Since then, there are reports of upregulation of CYP4A/4F enzymes in thyroid, breast, colon, and ovarian cancer, as well as pancreatic adenocarcinoma that all exhibit hyperactivated angiogenesis [7], and the therapeutic effects of 20-HETE inhibitors in these cancer types [84]. More recently, using oxygen–glucose deprivation of astrocytes and a mouse model of focal cerebral ischemia, Liu et al. reported that astrocyte-derived 20-HETE contributed to angiogenesis after the induction of ischemic stroke by inducing endothelial cell proliferation, tube formation, and migration [85].

In summary, the available data does not provide sufficient information to indicate whether 20-HETE is beneficial or detrimental to the prognosis of stroke. 20-HETE has shown a positive correlation with better recovery from stroke by promoting CBF autoregulation, reducing BBB leakage, and enhancing angiogenesis; it also possibly worsens stroke recovery by increasing endothelial dysfunction, oxidative stress, and vascular inflammation.

## 4. Conclusion and Perspectives

Hypertension is a common disease that puts millions at risk of developing stroke and other cardiovascular disorders. It is critical that patients are advised to address hypertension as soon as possible through lifestyle changes and pharmaceutical therapies so that renal damage and stroke risk can be mitigated. While hypertension is a modifiable risk factor for preventing stroke, there are definitive data showing that genetics also plays a major role. Certain SNPs are associated with hypertension and stroke in humans. In the kidney, 20-HETE protects against hypertensive damage through the constriction of Af-art. However, increased levels of 20-HETE are associated with fibrosis and renal damage. On the other hand, prolonged exposure to 20-HETE also has a pro-hypertensive role in the vasculature. Interestingly, 20-HETE also has a contradictory role in the brain. In the vasculature, reduced 20-HETE is associated with an impaired myogenic response, autoregulation, and BBB integrity. However, high amounts of 20-HETE have been found to cause endothelial dysfunction and activation as well as disruption of BBB, increasing the risk of stroke. 20-HETE is normally increased after ischemic and hemorrhagic strokes, and the use of 20-HETE inhibitors such as HET0016 during the early stages of strokes has been found to reduce infarct size, prevent vasospasm, and improve neurological outcomes. Because of the rise in 20-HETE post-stroke, the best outcomes are achieved using 20-HETE inhibitors in the early stages of stroke recovery. Moreover, 20-HETE plays an important role in neovascularization and angiogenesis that are necessary to stroke recovery; thus, more research needs to be done on the timing of therapy with 20-HETE inhibitors.

## Abbreviations

20-COOH-HETE	20-carboxy-eicosatetraenoic acid
20-HETE	20-hydroxyeicosatetraenoic acid
5-HT	serotonin
GWAS	Genome-wide association study
AA	arachidonic acid
ACE	angiotensin-converting enzyme
ACI	acute cerebral ischemia
ADH	alcohol dehydrogenase
Af-art	afferent arteriole
ANG II	angiotensin II
AT1	angiotensin II receptor 1
BBB	blood-brain barrier
BK	blocking calcium-activated potassium channel
CADASIL	cerebral autosomal dominant arteriopathy with subcortical infarcts and leukoencephalopathy
CARASIL	cerebral autosomal-recessive arteriopathy with subcortical infarcts and leukoencephalopathy
COX	cyclooxygenase
COX2	cyclooxygenase-2
CSD	cortical spreading depolarization
CSF	cerebrospinal fluid
CSI	cortical spreading ischemia
DCI	delayed cerebral ischemia
EETs	epoxyeicosatrienoic acids
EPC	endothelial progenitor cells
FAA	free fatty acids
FGF2	fibroblast growth factor 2
KO	knock out
LOX	lipoxygenase
MAPKs	mitogen-activated protein kinases
MCAo	middle cerebral artery occlusion
ND	neurological dysfunction
NHE3	sodium-hydrogen exchanger 3
NKCC2	Na-K-Cl cotransporter
NO	nitric oxide
PKC	protein kinase C
PLA2	phospholipase A2
PT	proximal tubule
RAAS	renin-angiotensin-aldosterone system
ROMK2	renal outer medullary potassium channel 2
SAH	subarachnoid hemorrhage
SHR	spontaneously hypertensive rat
SNP	single nucleotide polymorphisms
SS	salt sensitive
TALH	thick ascending loop of Henle
TBI	traumatic brain injury
TGF	tubuloglomerular feedback responses
TRPC6	transient receptor potential cation channel 6
TRPV1	transient receptor potential vanilloid 1
UGT	uridine 5’-diphosphoglucuronosyltransferase
VEGF	vascular endothelial growth factor
VSMC	vascular smooth muscle cell
WT	wild type
